# Age and Sex Impact Alzheimer’s Disease‐associated Plaques and Tangles in Down Syndrome

**DOI:** 10.1002/alz.091923

**Published:** 2025-01-03

**Authors:** Elizabeth J. Andrews, Michael J. Phelan, Phong Ngo, Alexis De La Rosa, Freddy Gonzalez, Jesse R. Pascual, Sierra Wright, Rachel F. Buckley, Gillian T Coughlan, Julia Kofler, Milos D Ikonomovic, Florence Lai, Beau Ances, Benjamin L Handen, Bradley T. Christian, Mark Mapstone, Elizabeth Head

**Affiliations:** ^1^ University of California, Irvine, Irvine, CA USA; ^2^ Department of Neurology, Harvard Medical School, Boston, MA USA; ^3^ Brigham and Women’s Hospital and Department of Neurology, Massachusetts General Hospital, Harvard Medical School, Boston, MA USA; ^4^ Massachusetts General Hospital, Harvard Medical School, Boston, MA USA; ^5^ University of Pittsburgh, Pittsburgh, PA USA; ^6^ Department of Neurology, Washington University at St. Louis, St. Louis, MO USA; ^7^ Waisman Center, University of Wisconsin‐Madison, Madison, WI USA; ^8^ The UC Irvine Institute for Memory Impairments and Neurological Disorders (UCI MIND), Irvine, CA USA

## Abstract

**Background:**

The prevalence of Alzheimer’s disease (AD) pathologies in people with Down syndrome (DS) is nearly 100%. In DS, overexpression of APP (on chr21) is associated with increased production of amyloid beta (Aβ) and the formation of phosphorylated tau (ptau) tangles. In the general population, women exhibit higher burdens of ptau compared to age‐matched men with AD. We hypothesized that in DS women would have higher ptau levels compared to men, and that age would significantly correlate with the presence of neuropathology.

**Methods:**

We examined 88 brains (frontal and occipital cortices) of people with DS (age 1‐39yr; M/F = 19/12) and DSAD (age 42‐61yr, M/F = 20/19) and age‐matched controls. Serial sections were immunostained for ptau (AT8) and Aβ (6E10) and quantified using annotated regions of interest from whole slide images in QuPath. Spearman rank correlations of load data were calculated, the Mann‐Whitney U test was used to compare men (n = 20) and women (n = 16) with DS. Mean positivity data was modeled using beta binomial regression in our cohort.

**Results:**

When comparing neuropathology loads of DS individuals (age 40+), women show consistently higher median levels of Aβ and ptau. Differences were not statistically significant. Aβ and ptau were both significantly correlated with age in the grey matter of the frontal (Aβ: r = 0.76, p≤0.001; ptau: r = 0.72, p≤0.001) and occipital cortices (Aβ: r = 0.72, p≤0.001; ptau: r = 0.70, p≤0.001). Interestingly, the positive correlation with ptau and age within the occipital cortex appears to be stronger in women (R = 0.80, p≤0.0001) than in men (R = 0.71, p≤0.01). We were able to determine that AD pathology presents in the frontal cortex approximately 10 years earlier than the occipital cortex and increases exponentially after age 40.

**Conclusion:**

These results suggest age significantly impacts AD pathology in DS, with neuropathologies presenting in the frontal cortex approximately 10 years earlier than the occipital. Trends indicate sex does appear to have an impact on these pathologies, particularly in the occipital cortex where ptau was found to have a stronger association with age in women. Collectively, this warrants further investigation on the impact of sex and age on AD neuropathology in DS and the contributing mechanisms.

